# Supplementary material for: PON-P3: Accurate Prediction of Pathogenicity of Amino Acid Substitutions
Source: Int J Mol Sci. 2025 Feb 25;26(5):2004. doi: 10.3390/ijms26052004 (PMC11899954; doi:10.3390/ijms26052004)
Supplement: Supplementary file 1 [file ijms-26-02004-s001.zip › ijms-3383239-supplementary.pdf]

PON-P3: accurate prediction of pathogenicity of amino acid substitutions
Supplementary material

Muhammad Kabir[1], Saeed Ahmed[1,2], Haoyang Zhang[1], Seyed Morteza Najibi[1,3], Ignacio Rodríguez-Rodríguez[1,4], Mauno Vihinen[1,*]

[1]Department of Experimental Medical Science, BMC B13, Lund University, SE-22184 Lund, Sweden

[2]Present address Department of Computer Science, University of Swabi, Swabi, Khyber Pakhtunkhwa 94640, Pakistan

[3]Present address Department of Clinical Medical Science, Section of Rheumatology, Lund University, Lund, Sweden

[4]Present address Departamento de Ingeniería de las Comunicaciones, Universidad de Málaga, Avda. Cervantes, 2, 29071 Málaga, Spain

Supplementary Table S1. Features used for training.

Feature	Dimension(s)
Protein and gene features	
Redundancy	1
Housekeeping	1
Essential gene	1
Haploinsufficiency	1
Complete knock-out gene	1
Lethality gene	1
Sequence length	1
Inheritance pattern	4
Protein-protein interaction network feature	9
Gene Ontology feature	3
Protein dipeptide composition	400
Gene age	1
Variation features	
Variation type	436
Neighborhood features	25
Variation in the first position	1
Amino acid properties	622
Relative position	1
PSSM	5
Pseudogene presence	1
Domain location	1
Repeat location	1
Structural features	
Secondary structural element	8
Accessibility	1
Transmembrane location	1
Intrinsically disordered region location	1
Total	1,526

Supplementary Table S2. Comparison of the effects of the number of trees on the performance of various classifiers in terms of 10-fold CV using all the features, except for the GO annotations. Two hundred bootstraps were applied in each case.

	LightGBM			RF			XGBoost		
Measure	200 trees[a]	250 trees	300 trees	200 trees	250 trees	300 trees	200 trees	250 trees	300 trees
Accuracy	0.934(0.926)	0.933(0.924)	0.932(0.923)	0.897	0.892(0.889)	0.889	0.934(0.927)	0.933(0.925)	0.933(0.924)
ΔAccuracy	0.426	0.424	0.422	0.390	0.389	0.384	0.427	0.425	0.424
MCC	0.866(0.857)	0.864(0.854)	0.861(0.851)	0.793	0.785(0.781)	0.779	0.866(0.858)	0.864(0.855)	0.864(0.854)
PPV	0.965(0.974)	0.966(0.975)	0.967(0.976)	0.921	0.917(0.928)	0.914	0.964(0.973)	0.966(0.975)	0.967(0.976)
NPV	0.915(0.887)	0.913(0.883)	0.91(0.88)	0.879	0.874(0.857)	0.871	0.914(0.889)	0.913(0.885)	0.913(0.883)
Sensitivity	0.875(0.875)	0.871(0.871)	0.867(0.867)	0.848	0.844(0.844)	0.842	0.879(0.879)	0.873(0.873)	0.87(0.87)
Specificity	0.977(0.977)	0.978(0.978)	0.979(0.979)	0.938	0.934(0.934)	0.931	0.975(0.975)	0.977(0.977)	0.979(0.979)
OPM	0.813(0.813)	0.81(0.81)	0.806(0.806)	0.721	0.711(0.711)	0.703	0.813(0.813)	0.81(0.81)	0.81(0.81)
Pathogenic as unknown	4990	4,996	4,936	4,222	3,971	3,744	4,696	4,871	5,005
Neutral as unknown	2449	2,388	2,357	2,829	2,631	2,513	2,364	2,362	2,366
TP	5,757(7,903)	5,722(7,916)	5,749(7,908)	6,232	6,410(7,466)	6,585	6,038(8,009)	5,845(7,957)	5,710(7,929)
TN	8,820	8889	8,926	8,113	8,264	8,345	8,890	8,910	8,917
FP	209	201	195	536	583	620	224	206	195
FN	820(1,126)	849(1,174)	882(1,213)	1,113	1,186(1,381)	1,238	833(1,105)	851(1,159)	852(1,183)
Coverage	0.677	0.68	0.684	0.694	0.714	0.728	0.694	0.686	0.68

[a]Numbers in brackets are for normalized cases. For each tool, the numbers of pathogenic variants were normalized to be equal to the number of benign variants. OPM, overall performance measure.
